# Application of the integrated rehabilitation-clinical-nutrition management model in geriatric rehabilitation patients: a narrative review

**DOI:** 10.3389/fpubh.2026.1831732

**Published:** 2026-06-19

**Authors:** Dongmei Mo, Yao Wang, Yongli Xu, Yingchao Guan, Ya-nan Gao, Daiwen Wang

**Affiliations:** Beijing Geriatric Hospital, Beijing, China

**Keywords:** clinical treatment, comprehensive management, geriatric rehabilitation, multidisciplinary collaboration, nutritional support, public health strategy

## Abstract

**Introduction:**

As the world's population ages, health problems among older rehabilitation patients show complicated features characterized by multimorbidity, functional decline, and interwoven dietary hazards.

**Methods:**

This study uses a narrative review methodology to systematically examine the theoretical framework, key components, and current practice of the integrated rehabilitation-clinical-nutrition management model among older rehabilitation patients.

**Results:**

The integrated management model demonstrates potential benefits in terms of functional recovery, nutritional improvement, prevention and control of complications, and mental health. The paper also critically examines the limitations of current research, evidence heterogeneity, and research gaps.

**Discussion:**

The collaborative integration-based integrated rehabilitation-clinical-nutrition management approach has emerged as a crucial tactic for boosting quality of life, optimizing healthcare resource distribution, and increasing functional outcomes among older patients. Suggestions for improvement are offered from the standpoints of public health service systems, human resource allocation, and policy support. This review aims to provide recommendations for clinical practice, health management, and policy-making, while encouraging more consistent and long-lasting use of integrated care models in geriatric rehabilitation.

## 1 Introduction

Population aging has become a global public health challenge. Aging is accompanied by multi-organ functional decline, reduced metabolic capacity, and weakened immune function, making older adults more susceptible to various chronic diseases, physical functional impairments, and malnutrition—collectively constituting the core manifestations of geriatric syndromes (1). These multidimensional health issues interact and create a vicious cycle, making it difficult for traditional single-discipline diagnostic and treatment models to achieve optimal rehabilitation outcomes. At the same time, the rising prevalence of noncommunicable diseases—such as stroke, ischemic heart disease, diabetes, and chronic obstructive pulmonary disease—further complicates geriatric rehabilitation management. Against this backdrop, the development of a collaborative, continuous, and patient-centered integrated health management model has become particularly urgent.

The Rehabilitation-Clinical-Nutrition Integrated Management Model is a holistic care model designed to address the complex needs of the geriatric rehabilitation population. Its core lies in the synergistic integration of three pillars: Rehabilitation Intervention: Based on functional assessment, this involves individualized exercise training, functional restoration, and enhancement of activity capacity; Clinical Treatment: Focusing on the control of underlying diseases, management of complications, medication safety, and disease stabilization; Nutritional Support: Focusing on nutritional screening, assessment, personalized interventions, and dynamic monitoring as key components. Unlike conventional multidisciplinary teams that provide simple parallel services, this integrated model emphasizes deep interdisciplinary collaboration, focusing on resolving the complex interactions among physical function, clinical conditions, and nutritional status in the older population. This review specifically concentrates on older adult rehabilitation patients in community settings, post-acute care, and those with chronic diseases, including primary application scenarios such as post-stroke rehabilitation, post-fracture rehabilitation, rehabilitation for chronic cardiopulmonary diseases, and care for frail and functionally declining older adult individuals. It does not extend its primary focus to populations outside geriatric rehabilitation contexts, such as ICU or perioperative surgical patients unless directly relevant to functional recovery in older adults.

Existing research indicates that in populations such as those undergoing critical care rehabilitation, perioperative surgical care, and intestinal failure, integrated multidisciplinary interventions can improve nutritional indicators, shorten hospital stays, and reduce the risk of complications (2–6). In the field of geriatric rehabilitation, integrated models have similarly demonstrated positive outcomes, such as optimizing post-stroke nutritional management, improving dysphagia, and promoting comprehensive recovery from post-intensive care syndrome (6–8). However, the current body of research on this specific model for geriatric rehabilitation remains fragmented. Studies often suffer from conceptual ambiguity regarding what constitutes true “integration,” heterogeneous intervention designs, and inconsistent outcome measurements, which collectively limit the strength of evidence and hinder translation into routine practice.

This article presents a narrative review to conduct a comprehensive analysis of the composition, implementation, outcomes, and challenges of integrated rehabilitation-clinical-nutrition management models for geriatric rehabilitation patients. It aims to address research limitations and evidence gaps, thereby providing a basis for clinical practice and policy optimization.

## 2 Methods

### 2.1 Review design

This is a narrative review. It aims to synthesize and critically appraise the existing literature on the integrated rehabilitation-clinical-nutrition management model in geriatric rehabilitation patients, without performing a systematic review or meta-analysis.

### 2.2 Literature search

We searched three electronic databases—PubMed, Web of Science, and the Cochrane Library—from inception to Mar 1, 2026. No language or publication date restrictions were initially applied. The search strategy combined keywords related to the population and the intervention. For PubMed, we used combinations of terms such as “geriatric rehabilitation”, “older adults”, “elderly”, and “aged” for the population, combined with “integrated model”, “multidisciplinary care”, “nutritional support”, and “rehabilitation-clinical-nutrition” for the intervention. The same core keywords were adapted for the other databases. We also manually screened the reference lists of relevant reviews and included original studies to identify additional eligible articles.

Study selection criteria (for guiding inclusion, not as a systematic protocol)

We included original clinical studies (randomized controlled trials, non-randomized controlled trials, cohort studies, before-after studies, and case series) as well as high-quality systematic reviews and clinical practice guidelines that met the following criteria:

- Population: Geriatric rehabilitation patients, defined as individuals aged 65 years or older receiving active rehabilitation for conditions such as stroke, hip fracture, cardiopulmonary diseases, or frailty in post-acute, community-based, or inpatient rehabilitation settings.
- Intervention: An integrated model combining at least two of the three core domains—rehabilitation (e.g., exercise, functional training), clinical treatment (e.g., disease management, medication optimization), and nutritional support (e.g., screening, individualized supplements) —delivered through multidisciplinary collaboration.
- Outcomes: Any measure related to functional recovery, nutritional status, complication prevention, mental health, or healthcare utilization/economic outcomes.
- Study type: Original research or systematic reviews published in peer-reviewed journals.

We excluded studies that:

- Focused exclusively on intensive care unit patients, perioperative surgical care without a rehabilitation phase, or long-term institutional care without active rehabilitation;
- Described a single intervention (e.g., nutrition alone or exercise alone) without multidisciplinary integration;
- Were conference abstracts, editorials, case reports, or animal studies.

### 2.3 Selection process and final set

Two authors independently screened titles and abstracts, then full texts. Disagreements were resolved through discussion. After removing duplicates, we identified 84 articles that met the selection criteria. These 84 articles form the primary evidence base for this narrative review.

### 2.4 Data synthesis and critical approach

Owing to the substantial heterogeneity across studies in design, populations, intervention components, outcome measures, and follow-up durations, we did not perform a meta-analysis or any formal statistical pooling. Instead, we conducted a thematic narrative synthesis organized by predefined themes: functional recovery and quality of life, nutritional status improvement and complication prevention, mental health and cognitive function, economic benefits, and implementation challenges.

Within each theme, we not only summarize key findings but also provide a critical appraisal of study limitations (e.g., single-center design, small sample size, short follow-up, confounding bias), transferability to routine geriatric rehabilitation practice, and evidence gaps. No formal risk-of-bias assessment tool (e.g., RoB 2.0, Newcastle-Ottawa Scale) was applied, consistent with the narrative nature of this review. Conclusions are framed cautiously, distinguishing between well-supported observations and preliminary or indirect evidence.

## 3 Theoretical foundations and core components of the rehabilitation-clinical-nutrition integrated management model

### 3.1 Multidisciplinary collaboration mechanism

Older adult rehabilitation patients often present with multiple comorbidities and concurrent functional and nutritional issues, requiring a collaborative team comprising rehabilitation physicians, clinicians, dietitians, nurses, and psychologists. Rehabilitation physicians coordinate rehabilitation goals and plans; clinicians manage underlying diseases, ensure medication safety, and prevent complications; dietitians conduct nutritional risk screening, provide dietary guidance, and implement individualized supplementation; nursing staff handle coordination, follow-up, and health education; and mental health professionals address the impact of emotional disorders on the rehabilitation process. A clear division of responsibilities within the team enhances is posited to team effectiveness and significantly strengthens team cohesion.

Multidisciplinary collaboration is typically achieved through regular case discussions, the establishment of rehabilitation goals and treatment plans, information sharing, and dynamic adjustments. However, in practice, issues such as communication barriers between disciplines, unclear delineation of responsibilities, and inconsistencies in assessment tools remain common, potentially affecting the continuity and consistency of integrated care (9–11). Critically, the evidence supporting the superiority of one specific team structure over another is weak. Most descriptions of multidisciplinary teams (MDTs) are based on expert opinion or qualitative reports, and there is a lack of rigorous comparative studies demonstrating how specific team processes directly impact objective patient outcomes in geriatric rehabilitation. The influence of local context, leadership quality, and team dynamics is likely substantial but remains under-researched. Establishing standardized processes, clarifying roles and responsibilities, and strengthening patient and caregiver involvement are logical steps that may help improve the efficiency of team collaboration.

### 3.2 Functional assessment and personalized rehabilitation design

Comprehensive functional assessment forms the foundation of geriatric rehabilitation. Commonly used tools include Activities of Daily Living (ADL), Instrumental Activities of Daily Living (IADL), cognitive assessments (MMSE, MoCA), the Functional Independence Measure (FIM), nutritional risk screening, and frailty assessments. Some studies suggest that FIM scores can guide home-based exercise rehabilitation for older adult stroke patients, and the resulting improvements in motor function and cognitive status have been reported as significantly superior to those achieved with conventional care (12). ADL independence is a strong predictor of post-stroke rehabilitation outcomes, and its importance may even surpasses that of certain cognitive learning potential assessments (13). Baseline functional status can to some extent indicate rehabilitation prognosis, and individualized plans based on multidimensional assessments are more targeted. With the development of wearable devices and remote monitoring technologies, functional assessment is gradually becoming dynamic and visualizable, providing objective evidence for adjusting rehabilitation plans. Incorporating psychosocial factors, family support, and environmental factors into the assessment aligns more closely with the holistic rehabilitation philosophy under the International Classification of Functioning, Disability and Health (ICF) framework (14). Nevertheless, the translation of comprehensive ICF-based assessments into practical, time-efficient clinical workflows remains a significant hurdle, and many studies linking assessment to outcome are observational in nature, unable to confirm a causal effect of assessment-driven personalization on long-term recovery. Future rehabilitation models should focus on utilizing standardized comprehensive assessment tools, combined with technological innovations to enhance accuracy and adaptability.

### 3.3 Integration of clinical treatment and rehabilitation

Older adult patients often have comorbid chronic conditions, necessitating the coordinated advancement of clinical treatment and rehabilitation training. Clinical interventions primarily focus on stabilizing the condition, controlling risk factors, and managing complications; rehabilitation interventions improve functional ability through exercise training, balance training, and respiratory training. The dynamic integration of these two approaches is intended to facilitates continuous care from the acute phase to the recovery phase and reduces the risk of adverse events. In the rehabilitation of cardiovascular diseases, chronic obstructive pulmonary disease (COPD), stroke, and musculoskeletal disorders, a model combining clinical indicator monitoring with adjustments to rehabilitation intensity has demonstrated potential advantages (15–19). However, most studies are observational or single-center, with small sample sizes and short follow-up periods, making it difficult to generalize findings to the broader geriatric rehabilitation population. Additionally, few studies have addressed how to balance clinical disease control with rehabilitation intensity in frail older adult patients, who are at higher risk of adverse events from overly aggressive rehabilitation. The integration of clinical care and rehabilitation embodies a patient-centered philosophy, balancing disease management with functional recovery, and is particularly well-suited to the needs of an aging society. The concept is compelling, but the operational details and proof of long-term efficacy are still developing.

### 3.4 The critical role of nutritional support in geriatric rehabilitation

Malnutrition, sarcopenia, and impaired swallowing function are prevalent among older adult rehabilitation patients, potentially delaying muscle repair, compromising immune function, and increasing the risk of complications (20, 21). Early nutritional screening and individualized interventions—including protein supplementation, vitamin D optimization, and oral nutritional supplements—may help improve skeletal muscle mass, enhance immune function, and promote functional recovery (22). Dynamic nutritional adjustments led by dietitians and the multidisciplinary Nutrition Support Team (NST) model have been shown to significantly improve rehabilitation outcomes and quality of life in postoperative and chronic disease patients, demonstrating positive clinical value (23, 24). However, significant individual variability among older adults, high rates of dysphagia, and inconsistent nutritional adherence limit the effectiveness of standardized nutritional protocols. Furthermore, many geriatric rehabilitation settings lack dedicated dietitians, leading to suboptimal nutritional intervention implementation, and there is insufficient evidence to determine the optimal timing and dosage of nutritional supplements for frail older adult rehabilitation patients. Future efforts should focus on refining nutritional assessment tools, optimizing supplementation regimens, and conducting in-depth investigations into the mechanisms underlying the interactions between nutrition, exercise, and immune function.

## 4 Current status and practical cases of the integrated rehabilitation-clinical-nutrition management model

### 4.1 Comparative analysis of implementation status domestically and internationally

Internationally, countries with more advanced aging populations have long prioritized the integration of geriatric rehabilitation and nutrition, emphasizing early assessment, multidisciplinary collaboration, and continuous community-based services (25, 26). Many developed countries are increasingly recognizing nutritional intervention as a core component of geriatric rehabilitation. In China, with the development of Enhanced Recovery After Surgery (ERAS), the establishment of geriatric medicine centers, and the improvement of the rehabilitation healthcare system, integrated models are gradually being applied in settings such as stroke, fracture, and cardiopulmonary rehabilitation (27). However, several common challenges persist: insufficient staffing of dedicated dietitians, inconsistent implementation rates of nutritional screening, loose integration between rehabilitation and nutrition, and significant regional disparities in implementation. Future efforts should focus on standardizing clinical protocols, expanding the professional workforce, and promoting seamless integration of nutrition and rehabilitation to effectively address the complex needs of an aging population.

### 4.2 Analysis of typical clinical practice models

This paper categorizes integrated models according to clinical dimensions such as functional rehabilitation, nutritional management, and technical support, with a particular focus on older adult rehabilitation populations. In cardiac rehabilitation, post-fracture rehabilitation, and stroke rehabilitation, these models have become widely adopted and have demonstrated significant efficacy. In cardiopulmonary rehabilitation, the coordination of clinical medication management, respiratory training, and nutritional support helps improve exercise tolerance and enhance quality of life (28). In fracture and musculoskeletal rehabilitation, early rehabilitation training combined with nutritional optimization may promote bone healing, improve balance, and reduce the risk of re-fall (10, 29). In stroke rehabilitation, the integration of exercise training, swallowing management, nutritional support, and cognitive interventions helps improve functional independence and reduce complications (30, 31). In frailty and functional decline rehabilitation, based on multidimensional assessments, progressive training, nutritional fortification, and psychological support are used to delay functional decline. Current practice suggests that a typical clinical model of integrated rehabilitation-clinical-nutrition management, through multidisciplinary collaboration, patient-centered care, and resource integration, can effectively improve functional recovery, nutritional status, and quality of life in older adult patients. Continued strengthening of clinical physician training and the development of standardized protocols will further optimize such integrated care models; however, it is also noted that the effectiveness of these integrated models is closely related to team collaboration efficiency, patient compliance, and the availability of resources.

### 4.3 Application of information technology in integrated management

The integration of information technology has significantly improved the quality and efficiency of the rehabilitation-clinical-nutrition integrated management model. Electronic health records, telerehabilitation platforms, wearable devices, and smart monitoring systems support multidisciplinary information sharing, home-based rehabilitation, and dynamic follow-up (32–36). Digital tools play a key role in enhancing care coordination, enabling personalized interventions, and improving clinical outcomes to a certain extent; however, issues such as varying levels of digital literacy among older adults and insufficient device compatibility continue to limit their widespread adoption.

### 4.4 Patient and family education and support

Health education, psychological support, and family involvement play a crucial role in improving rehabilitation adherence. Studies have confirmed that educational attainment and levels of social support directly influence patients' ability to follow rehabilitation protocols (37). Health education programs cover disease knowledge, medication management, nutritional guidance, rehabilitation exercises, and emotional regulation, utilizing diverse methods such as face-to-face counseling, printed materials, group sessions, digital platforms, and telemedicine (38–41). Diverse educational approaches are essential for improving adherence, functional recovery, and quality of life; however, many health education programs are not tailored to the needs of older adult rehabilitation patients: materials may be too complex, delivery methods may not accommodate physical or cognitive limitations, and family caregivers—who often play a key role in supporting rehabilitation—are not always included in educational interventions. Additionally, there is insufficient evidence to determine which educational methods are most effective for different subgroups of older adult rehabilitation patients, such as those with cognitive impairment or low health literacy. Continuous optimization of educational content, delivery methods, and technology integration is still needed to meet the complex and evolving needs of older adult rehabilitation patients.

## 5 Effectiveness evaluation and clinical value of the rehabilitation-clinical-nutrition integrated management model

### 5.1 Functional recovery and quality of life improvement

Existing research suggests that an integrated rehabilitation-clinical-nutrition management model significantly improves motor function, balance, and activities of daily living in older adult patients, with positive trends observed in the rehabilitation of stroke, fractures, and cardiopulmonary diseases (42–46). Notably, the results of rehabilitation differ from person to person. For example, older female cardiac rehabilitation patients showed significant functional improvement, but they also had more severedepression symptoms, which required specific psychological interventions (47). Currently, most studies have limited sample sizes, short follow-up periods, and lack standardized outcome measures; therefore, conclusions should be interpreted with caution. However, based on current research findings, developing personalized intervention plans tailored to disease type, functional status, and psychosocial factors, and optimizing rehabilitation pathways, can facilitate older adults' reintegration into society and ultimately improve their overall quality of life.

### 5.2 Nutritional status improvement and complication prevention

Nutritional support combined with rehabilitation training is crucial for enhancing the overall health of older adult rehabilitation patients, with particularly significant effects on improving body weight, muscle mass, and immune function. Studies have confirmed that such interventions can, to a certain extent, elevate albumin levels, increase muscle mass, reduce the risk of infection and pressure ulcers, and shorten length of stay (48–53). Economic evaluations further support the value of nutritional management: malnutrition is associated with increased readmission rates and prolonged length of stay (48). These economic benefits reinforce the rationale for integrating nutritional management into comprehensive rehabilitation care. Additionally, the synergistic effects of nutritional management and rehabilitation measures can promote muscle preservation and enhance immune resilience, which are crucial for managing sarcopenia and frailty. Early, individualized nutritional interventions hold particular potential value for postoperative, frail, and high-risk older adult patients, making an integrated rehabilitation-clinical-nutrition management model an essential measure for optimizing geriatric rehabilitation.

### 5.3 Impact on mental health and cognitive function

According to some research, psychological distress partially mediates the relationship between cognitive function and physical frailty in older adults, and the integrated rehabilitation-clinical-nutrition management model is very successful in reducing depression, anxiety, and cognitive impairment in older adult patients (54). A model that integrates psychological support, nutritional intervention, and rehabilitation training may help alleviate anxiety and depression and improve cognition-related indicators (55–58). Research has shown that increased sedentary behavior and decreased physical activity are directly linked to depressive symptoms; nutritional status is significantly associated with cognition and depression. Developing psychological resources like dispositional optimism might mitigate the effects of sensory impairments on depressive symptoms (55, 59). However, there remains a notable scarcity of high-quality, large-sample, long-term follow-up controlled studies specifically designed to test the mental health and cognitive benefits of an integrated model. The existing evidence is largely indirect, derived from studies of individual components. Future trials with mental health and cognitive outcomes as primary endpoints are needed to confirm the synergistic effects currently inferred from component-specific evidence.

### 5.4 Economic benefits and healthcare resources utilization

Among older patients, the integrated rehabilitation-clinical-nutrition treatment strategy has shown notable financial advantages. Quality-adjusted life years (QALYs) are frequently used as a core indicator in cost-effectiveness assessments because they show both economic gains and increases in quality of life, making it easier to compare various interventions (60). Extending stroke rehabilitation services was confirmed by a randomized controlled experiment. Randomized controlled studies found that while extending stroke rehabilitation treatments greatly increased patient and caregiver satisfaction, it had no discernible effect on patients' everyday activities. This approach achieved a 90% likelihood of cost-effectiveness, raised 0.07 QALYs, and generated an average savings of £311 per case (61). For older female patients with vertebral fractures, home-based exercise regimens had an incremental cost-utility ratio of CAD 29,650 per QALY, with fewer side events offsetting initial costs (62). With incremental cost-utility ratios of £12,817 per QALY—within acceptable criteria—modified-texture meals and home enteral nutrition demonstrated significant cost-effectiveness in treating post-stroke dysphagia (63). This approach produces advantages that go beyond the individual level in addition to optimizing healthcare resources. For instance, the Mobile Integrated Care program decreased overall costs per thousand calls and unnecessary emergency transfers by 58–59% (64). The REACT trial, which demonstrated that community-based physical activity programs gained 0.04 QALYs and created a net savings of £103 per person, confirmed the economic viability of community-based interventions (60). Therefore, lowering healthcare expenditures, readmission rates, hospital stays, and complication-related charges are the main ways this approach achieves a certain degree of cost-effectiveness.

## 6 Challenges and improvement strategies for the rehabilitation-clinical-nutrition integrated management model

### 6.1 Barriers to multidisciplinary team formation and collaboration

Establishing a multidisciplinary team encompassing geriatric rehabilitation, clinical care, and nutrition is crucial; however, differences in the professional backgrounds of team members often lead to communication barriers, unclear delineation of responsibilities, and inconsistent workflows. For example, despite practitioners' positive attitudes toward collaboration, a study on multidisciplinary teams in Jakarta nursing homes found that optimal care coordination was hampered by professionals' lack of shared responsibility (65), underscoring the urgency of removing communication barriers and defining team roles. To address this challenge, enhancing team training and standardizing processes are key strategies. Team cohesion can be increased by training programs based on team science principles that emphasize mutual respect and cooperation skills (66). Information sharing and accountability are facilitated by the use of standardized workflows and communication tools, such as organized multidisciplinary team meetings and shared documentation platforms (67, 68). Team effectiveness is also greatly impacted by managerial support and leadership. Open communication and collaborative decision-making are fostered by effective leaders (69). By fostering psychologically secure environments that promote members' emotional wellbeing and dispute resolution, leadership in palliative care settings improves communication efficiency (70); without proactive leadership, teams may face issues such as high staff turnover and siloed work practices. These elements collectively create a collaborative environment that benefits patient outcomes; however, customized plans must be developed in accordance with local conditions to optimize team operations and enhance integrated healthcare services.

### 6.2 Implementation challenges of personalized nutritional interventions

Older adult patients exhibit high heterogeneity in nutritional needs; factors such as dysphagia, low nutritional literacy, and fluctuating adherence all impact intervention outcomes (71, 72). Dysphagia not only affects food intake but also increases the risk of aspiration pneumonia, a leading cause of death among the older adult in long-term care facilities (72); Patients with chronic illnesses typically have low and highly fluctuating nutritional literacy, which makes it difficult for them to follow their own dietary plans (73). Together, these elements highlight how important it is to create customized plans. The limitations of current nutritional assessment tools complicate implementation; traditional methods such as body mass index, serum albumin levels, or brief nutritional assessment scales may fail to comprehensively capture the multiple influencing factors in older adult patients (21, 74, 75); although inflammatory markers have been proven to be key predictors of postoperative pain prognosis, their clinical application remains limited due to resource constraints; this gap underscores the need to develop multidimensional nutritional assessment tools. Insufficient staffing of dietitians further limits standardized implementation. Given these challenges, a multidimensional strategy is required. Key to overcoming implementation hurdles lies in conducting precise nutritional assessments, adopting a patient-centered approach, actively integrating nutrition experts into collaborative care models, combining traditional and emerging diagnostic tools, addressing compliance barriers, and leveraging the professional expertise of nutritionist.

### 6.3 Insufficient resource allocation and policy support

Healthcare institutions face challenges such as staff shortages, inadequate infrastructure, and insufficient funding. Research reveals issues with community health-social partnership programs, such as unclear team responsibilities and inadequate resource support (76, 77); advanced assessment equipment is frequently lacking or out-of-date; healthcare accessibility for the older adult is impacted by financial constraints; and factors like chronic diseases and functional impairment influence expenditures (78). However, policy support is still insufficient and uneven. Although some areas have successfully adopted community-based health-social cooperation projects (77), The standardization and widespread promotion of integrated care models are hampered by the lack of a clear policy framework. Inadequate coordination between clinical and nutritional services, fragmented care services, and regional variations in results are caused by the absence of specialized policies (76). Given these current challenges, future efforts must establish a policy framework that explicitly prioritizes comprehensive geriatric rehabilitation and nutrition management, supported by sustainable funding mechanisms. Structured, evidence-based interdisciplinary teams can improve treatment outcomes and reduce the use of unplanned medical services; strengthen talent development by training more rehabilitation and nutrition professionals; promote cross-sectoral collaboration to integrate infrastructure and community resources, thereby maximizing service delivery efficiency; Continuously evaluate and adjust policies based on emerging evidence to ensure an effective response to the evolving needs of the older population and enhance sustainability.

### 6.4 Improving evaluation systems and follow-up mechanisms

The practical usability and scientific validity of current assessment markers for patients undergoing geriatric rehabilitation continue to be crucial concerns. Adverse outcomes, including postoperative hospitalization, falls, and mortality, have been successfully predicted by instruments such as multidimensional prognostic indices, short physical function tests, grip strength assessments, and geriatric depression ratings (12, 19, 44, 79). Rehabilitation is a dynamic and continuous process; establishing long-term follow-up and dynamic adjustment mechanisms is crucial for maintaining rehabilitation efficacy (80, 81). However, current assessment indicators are relatively scattered, and systems for dynamic monitoring and long-term follow-up remain underdeveloped, making it difficult to comprehensively evaluate the long-term effects of integrated models. In addressing this issue, information technology offers significant potential for enhancing assessment and follow-up. Virtual reality technology offers objective real-time data on functional status and compliance (82), telemedicine and digital health platforms enable synchronous remote precision adjustments and follow-up (67, 83), and internet-based rehabilitation management systems enhance the physical and psychological recovery outcomes of older adult hip fracture patients (81, 84). Therefore, utilizing information technology to build continuous follow-up systems and promote standardized assessment tools represents an important direction for future improvement.

### 6.5 Major limitations of the current evidence

Most of the literature retrieved to date consists of observational, single-center, small-sample studies, with few randomized controlled trials. Research designs are limited, and the level of evidence is restricted. Most studies focus on short-term functional and nutritional changes, with relatively short follow-up periods; data on long-term outcomes such as falls, readmissions, mortality, and quality of life are relatively scarce. There are significant differences in the composition, intensity, and implementation processes of integrated models, and the heterogeneity of interventions is pronounced, making direct comparison and generalization difficult. Current research primarily targets older adult rehabilitation patients in community settings, post-acute care, and those with chronic diseases; there are few targeted studies on specific older adult subgroups such as the very older adult, frail individuals, those with dementia, and those with severe disabilities. To address these shortcomings, the conduct of multicenter, prospective, randomized controlled trials is encouraged to elevate the level of evidence. Extend the follow-up period and standardize outcome measures by extending follow-up to 12 months or longer, systematically collecting long-term outcomes such as falls (frequency/severity), readmission rates, all-cause mortality, and quality of life to establish a standardized set of outcomes. Develop reporting guidelines for integrated rehabilitation interventions, detailing the components of the intervention, intensity (frequency/duration/cycle), personnel, location, adherence, and reasons for adjustments. This will reduce intervention heterogeneity and facilitate comparisons between studies and meta-analyses. Strengthen multicenter collaboration and data sharing by establishing regional or national geriatric rehabilitation networks. Standardize data collection platforms and share individual-level data to facilitate personalized meta-analyses and investigations into sources of heterogeneity. Furthermore, and most importantly, prioritize health economic evaluations. In addition to assessing clinical efficacy, concurrently conduct cost-effectiveness or cost-utility analyses to provide economic evidence for health insurance reimbursement and policy implementation.

## 7 Implications of the rehabilitation-clinical-nutrition integrated management model for public health practice and policy

From a public health perspective, the integrated rehabilitation-clinical-nutrition management model is of great significance for addressing the health challenges of an aging population and improving the efficiency of health services. This model targets older adult rehabilitation patients in the community, as well as those in the post-acute and chronic disease phases. However, community and primary care institutions currently lack rehabilitation and nutrition professionals, and mechanisms for inter-institutional referrals and continuous care are inadequate, making it difficult to implement this integrated model at the grassroots level. Given the substantial human resource needs, it is essential to strengthen interdisciplinary training for rehabilitation physicians, clinical dietitians, geriatric specialty nurses, and general practitioners to enhance the team's capacity for integrated care. It is recommended to establish a comprehensive service system and build a geriatric rehabilitation network that bridges “hospital–community–home,” incorporating nutritional screening, functional assessment, and chronic disease management into basic public health services. At the same time, the implementation of policy support should be encouraged, including the incorporation of integrated rehabilitation programs into medical insurance and long-term care insurance coverage, as well as the improvement of performance evaluation mechanisms to guide the allocation of medical resources toward geriatric health management and functional maintenance. Through systematic development, the integrated model can not only improve individual rehabilitation outcomes but also help alleviate the burden of social care and enhance the effectiveness of public health services.

## 8 Conclusion

Multidisciplinary cooperation is at the heart of the integrated rehabilitation-clinical-nutrition management concept. It offers comprehensive care that encompasses the concepts of precision and individualized medicine by addressing the interconnectedness of functional, nutritional, and clinical concerns in aged people. A road toward a more patient-centered approach is provided by the present literature, which indicates substantial clinical and financial promise in enhancing mobility, optimizing nutritional status, and possibly lowering the risk of problems. But the generation of evidence in this sector is still in its infancy. Conclusions concerning its efficacy are limited by ongoing issues with team implementation, resource limitations, and the absence of defined evaluation methodologies. Standardizing implementation processes, bolstering multidisciplinary training, and creating reliable information-based follow-up mechanisms are all crucial for the future. This strategy has the potential to improve the sustainability and quality of geriatric rehabilitation management and help alleviate the demands on public health caused by population aging through thorough research and supporting legislation.

## Data Availability

The original contributions presented in the study are included in the article/supplementary material, further inquiries can be directed to the corresponding author.
